# Case Report: Spontaneous Remission of an Infraorbital Follicular B-Cell Lymphoma: Case Report and Review of the Literature

**DOI:** 10.3389/pore.2021.642433

**Published:** 2021-04-08

**Authors:** Maxime Peeters, Joris Geusens, Fréderic Van der Cruyssen, Lucienne Michaux, Laurence de Leval, Thomas Tousseyn, Peter Vandenberghe, Constantinus Politis

**Affiliations:** ^1^ Department of Oral and Maxillofacial Surgery, University Hospitals Leuven, Leuven, Belgium; ^2^ OMFS-IMPATH Research Group, Department of Imaging and Pathology, Faculty of Medicine, Catholic University Leuven, Leuven, Belgium; ^3^ Department of Human Genetics, University Hospitals Leuven, Leuven, Belgium; ^4^ Department of Pathology, Lausanne University Hospital (CHUV) and Lausanne University, Lausanne, Switzerland; ^5^ Department of Pathology, University Hospitals Leuven, Leuven, Belgium; ^6^ Department of Hematology, University Hospitals Leuven, Leuven, Belgium

**Keywords:** follicular lymphoma, cheek, spontaneous remission, head and neck neoplasms, case reports

## Abstract

Non-Hodgkin lymphomas comprise a heterogeneous group of malignancies, with a wide scope of clinical, radiological and histological presentations. In this paper, a case is presented of a 59-year-old white male with an infraorbital follicular B-cell lymphoma, which appeared as a painless mass in the left cheek. The lymphoma achieved spontaneous remission five and a half months after his diagnostic incision biopsy. The literature is reviewed, focusing on this rare site of presentation and spontaneous remission. In literature, only four cases have been reported with a follicular B-cell lymphoma of the cheek or infraorbital region, and only 26 cases of spontaneous remission of an extracranial non-Hodgkin lymphoma in the head and neck region have been described. To the authors’ best knowledge, this is the first time spontaneous remission of an infraorbital follicular lymphoma could be observed. The nature of the processes inducing spontaneous remission remains obscure. It is important to recognize this phenomenon as this might prevent unnecessary treatment.

## Introduction

Non-Hodgkin lymphomas (NHL) comprise a heterogeneous group of malignancies, generally consisting of T-cell and B-cell neoplasms. Follicular lymphoma, a NHL, is the most common indolent lymphoma and accounts for around 10–20% of all lymphomas in western countries [1].

Spontaneous remission of cancer is a rare and intriguing phenomenon, with an unknown underlying mechanism, still being a subject of debate for clinicians, radiologists, pathologists, hematologists and researchers. Spontaneous remission appears in one case per 80,000–100,000 and occurs sporadically in patients with NHL [2].

The cheek and infraorbital area are uncommon sites for lymphomas. Spontaneous remission of follicular lymphoma located in this area is even more exceptional and has never been reported to our knowledge. This paper describes a well-documented case of a patient diagnosed with an infraorbital localized follicular lymphoma, following spontaneous remission within months.

## Case Presentation

**FIGURE 1:**
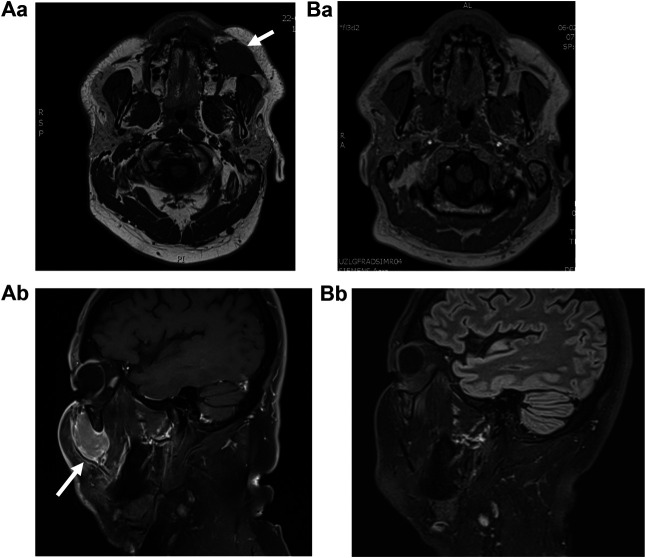
MRI (Magnetic Resonance Imaging) **(A)** at the moment of first presentation, depicting a volume of 28 mm × 29 mm × 42 mm (AP × ML × CC) and **(B)** 5.5 months later, after an incision biopsy was performed. Aa, This T2 weighted image depicts a well-described, mass of intermediate intensity at the buccinator space. Ba, T2 weighted image depicting the absence of the lesion, indicative for tumor remission. Ab, T1 weighted image with Gadolinium contrast, showing expansion to the infraorbital region. Contrastcaptation is moderate. Bb, T1 weighted image depicting the absence of the lesion.

A 59-year-old white male was referred to the University Hospitals Leuven, Department of Oral and Maxillofacial Surgery for evaluation of a left infraorbital mass. The mass presented two months earlier as a small nodule, rapidly increasing in size over a period of two days. Because the swelling persisted and even increased in size, the patient consulted his general practitioner. Consecutively, an ultrasound and a Magnetic Resonance Imaging (MRI) scan (Figure 1A) of the head and neck were performed. The patient was referred to the Oral and Maxillofacial Surgery Department, as these investigations suggested a schwannoma of the left infraorbital nerve.

**FIGURE 2:**
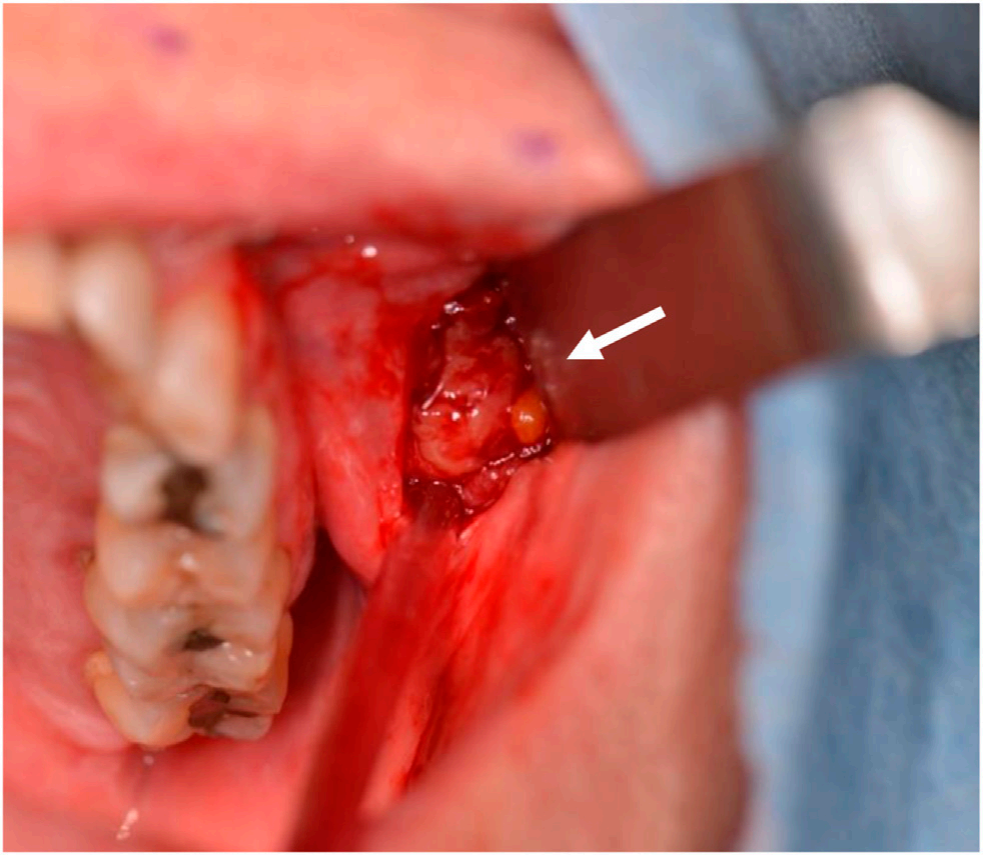
Biopsy. The mass presented as a well-described, encapsulated lesion.

The patient’s medical history consisted of recurrent vestibulopathy, vitreous floaters, protein S deficiency, and two episodes of popliteal vein thrombosis, in 1999 and 2006. He was taking Atorvastatin and Rivaroxaban daily. B symptoms were absent. Clinical examination revealed an infraorbital, mobile mass measuring 4 × 2 cm **(Appendix 1: PDF VECTRA)**. Quantitative Sensory Testing indicated a very subtle hypo-aesthesia of the left infraorbital nerve. Laboratory testing showed no abnormalities. An incision biopsy (0.8 × 1 cm) was performed under local anesthesia. During this procedure, a well-described, encapsulated mass was encountered (Figure 2).

**FIGURE 3:**
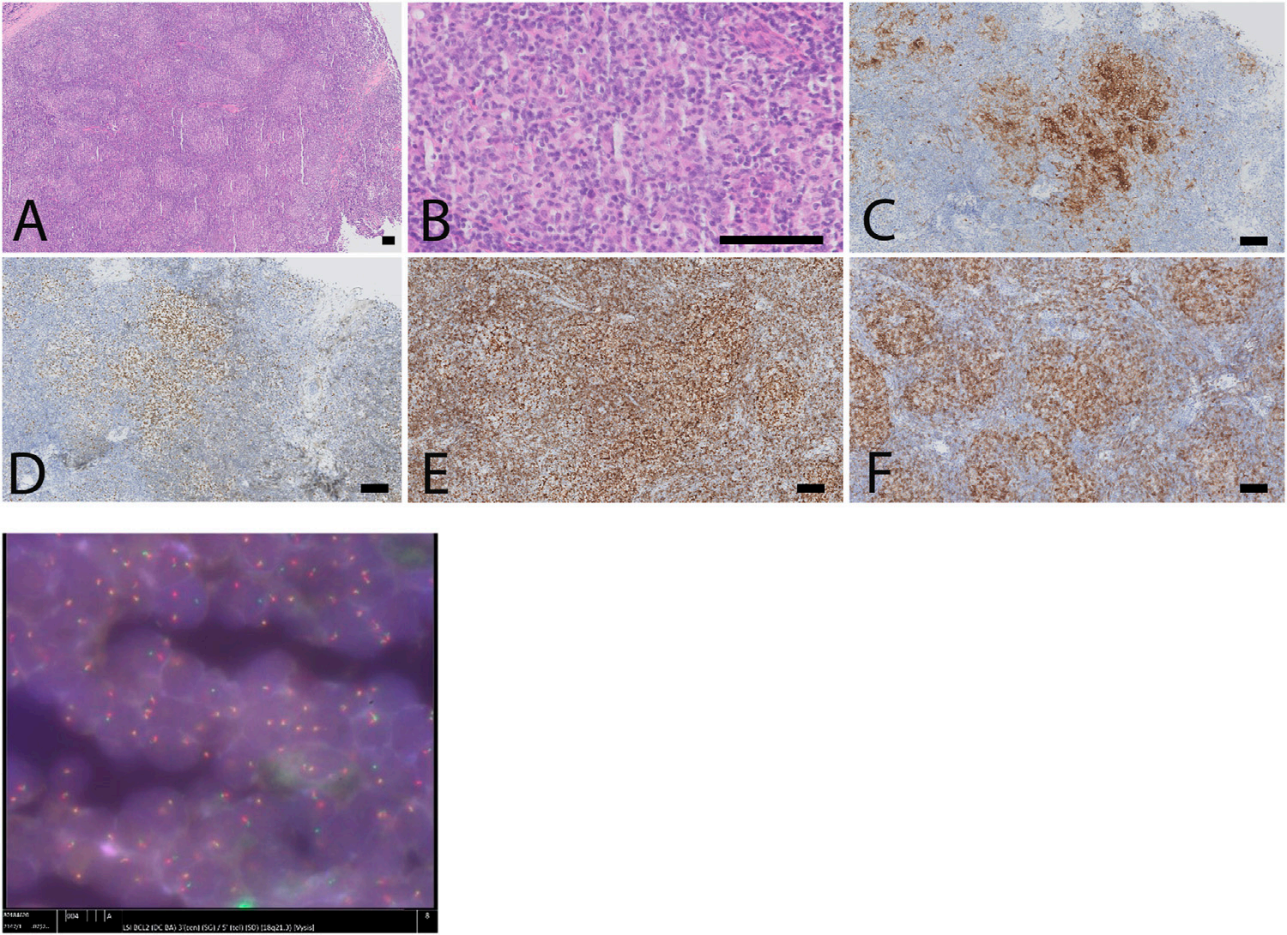
Lymph node biopsy, showing a follicular lymphoma with brisk TFH response and *BCL2*-rearrangement. **(A)** low power view showing a disturbed lymph node architecture, with numerous small follicles arranged in a back-to-back fashion. **(B)**. The follicle centers are composed predominantly of small centrocytes and scattered centroblasts (less than 15/high power field), in the absence of tangible body macrophages. The B-cells in de follicles express CD10 **(C)**, BCL6 **(D)**, overexpress BCL2 **(E)** and are intermingled with numerous follicular T-helper cells, as illustrated by a immunostaining against PD1 **(F)**. **(G)** FISH highlights the presence of a BCL2-rearrangement, corresponding to the BCL2 overexpression in the follicles, as illustrated in the anti-BCL2 immunostain **(E)** (scale bar: 100 µm).

Histological assessment was performed by the Pathology Department of University Hospitals Leuven, and reviewed by the Pathology Department of Lausanne University Hospital (CHUV). It showed a disturbed lymph node architecture, with vague nodules organized in a back-to-back manner (Figure 3). The follicles were composed of a mixture of centrocytes and centroblasts (<15/HPF), and numerous small T-lymphocytes, but no tingible body macrophages. The B-cells in the nodular infiltrates expressed CD20^+^, PAX5^+^, CD10^+^, Bcl6^+^, Ki 67^+^ (<20%) and overexpressed BCL2. The nodules were supported by CD21^+^ follicular dendritic networks. In between the B-cells in the follicle centers, there were increased numbers of CD3^+^, PD1^+^, CXCL13^+^, weak ICOS^+^ follicular helper T-cells. Both heavy and light chain gene rearrangements, as well as T-cell receptor beta and gamma gene rearrangements were found. FISH, using probe BCL2 (DC BA)[18q21, Vysis], uncovered a rearrangement of locus *BCL2*/18q21 in the B-cells. These findings yielded the diagnosis of a B-cell follicular lymphoma, grade 1-2, BCL-2 positive, with *BCL2*-gene rearrangement, with numerous intrafollicular follicular T-helper cells. The florid TFH cell (T-follicular helper cell) infiltrate is unusual but we would favor that it is reactive in nature. The presence of small T-cell clones in the presence of B-cell lymphomas are likely indicative of a prominent clonal component in the tumor, and do not warrant a diagnosis of composite follicular T-cell lymphoma.

Staging included clinical examination, and an 18F-fluorodeoxyglucose (FDG) positron emission tomography (PET) computed tomography (CT) scan, and revealed no other lymphadenopathies, nor FDG-avid lesions. Because of technical problems, the CT scan generated unusable images. As the infraorbital hypo-aesthesia persisted, further imaging was ordered which revealed a complete and spontaneous remission of the tumor mass (5.5 months after the initial MRI) (Figure 1B). Clinical examination was consistent with these findings. Bone marrow trephine and aspirate were negative. The tumor showed no recurrence during clinical and biochemical follow-up, six months after its spontaneous remission. The patient will receive a clinical and biochemical follow-up after one year.

## Discussion

Malignant lymphoma accounts for 5% of all head and neck cancers and an estimated 2.5% of all malignant lymphomas arise at the level of the cheek [3, 4]. Follicular lymphoma of the cheek mostly occurs as a parotid gland tumor [3, 5]. Apart from this parotid localization, follicular lymphomas are very rare at the level of the cheek. To the authors’ best knowledge, only four cases of a non-salivary gland, infraorbital or buccal localization of follicular lymphoma are reported in English literature (Table 1).

**TABLE 1:** Summary of reported cases of follicular lymphoma of the cheek or infraorbital region.

Case no	Author	Year	Sex/age	Tumor	Localization	Treatment	Follow-up
1	Farquhar et al. [25]	2014	F/66 Y	Follicular lymphoma	Cheek	Steroids for unrelated asthma, with spontaneous remission of the tumor	2 W
2	Hardin et al. [26]	2018	M/71 Y	Follicular lymphoma	Cheek, skin	—	—
3	Dhadlie et al. [27]	2018	F/70 Y	Follicular lymphoma, low-grade	Cheek, subcutaneous	—	—
4	Thaker et al. [28]	2019	F/65Y	Follicular lymphoma, low-grade	Cheek, superficial to the masseter muscle and parotid gland	Surgical excision	—
Present case	Peeters et al.	2020	M/59 Y	Follicular lymphoma	Buccal space to canine fossa	—	5.5 M

F, female; M, male; W, week; M, month

Lymphomas can originate nodal as well as extranodal. In this case, the presentation and histological findings indicate a nodal localization as 1) lymph nodes are situated in the canine fossa and buccinator space, referred to as ‘forgotten lymph nodes’; 2) an isolated infraorbital lymphoma most frequently arises in the infraorbital lymph node; 3) follicular lymphomas occur more often nodally (76.6%); and 4) BCL-2 expression and rearrangement of locus *BCL-2*/18q21 indicate a nodal origin rather than an extranodal origin in follicular lymphomas [6–11].

Lymphomas are a heterogeneous group of malignancies with a heterogeneous clinical presentation, often mimicking other malignant and benign lesions. Lymphoma is therefore considered as a ‘great imitator’ [12]. Nodal lymphoma has heterogeneous radiologic features as well [13]. In this case report, the patient presented with a painless mass in the cheek with a very subtle hypo-aesthesia, highlighting the indolent nature of follicular lymphomas [14]. Infraorbital and buccal masses have a variety of etiologies, e.g. odontogenic infection, salivary gland tumor, inflammatory lesion, lipoma, or schwannoma [15, 16]. Due to its acute development, an infectious process was suspected.

Follicular lymphoma is indolent and has a good prognosis, albeit it mostly remains an incurable disease [14]. In this case, it was diagnosed at Ann Arbor stage IA which is rather unusual. Prognosis depends on staging and risk stratification. The Follicular Lymphoma International Prognostic Index score (FLIPI) is widely used to assess the outcome of follicular lymphoma and includes five prognostic factors: age, stage, number of involved nodal areas, serum lactate dehydrogenase and hemoglobin. PET-CT is the standard of care for initial evaluation and staging of lymphoma. PET-CT identified no FDG-avid lesions in this case report. Non-FDG-avidity is associated with indolent lymphomas, and occurs in around 5% of all follicular lymphomas [17]. As the diagnostic lesion did not display FDG-avidity, other associated lesions might have been non-FDG-avid as well. PET detects more nodal sites than CT does, yielding a moderate up-staging in 11% of the nodal follicular lymphomas only assessed by CT; non-FDG-avidity might, therefore, lead to a down-staging of the FLIPI score [18]. In these cases, FLIPI-2 (age, β_2_‐microglobulin, diameter lymph node, bone marrow involvement and hemoglobin) or PRIMA-PI (β_2_‐microglobulin, and bone marrow involvement; for *de novo* follicular lymphoma treated initially with immunochemotherapy) are useful prognostic measures, as they do not include PET results [19, 20].

### Spontaneous Remission

Spontaneous remission of cancer is a well-documented, rare phenomenon. The underlying mechanism is still poorly understood and appears to be multifactorial [2]. Several hypotheses might explain the spontaneous remission of cancer, unified by the idea that the innate immune system is triggered and increases its ability to recognize and react on malignant cells [21]. Spontaneous remission occurs mostly in melanomas, hypernephromas and neuroblastomas, and might occasionally take place in lymphomas [22, 23]. To the author’s best knowledge, 26 cases of extracranial, head and neck NHL with spontaneous remission have been reported in English literature (Table 2; excluding relapsing lymphomas and cases that arise in a context of post-transplantation or immunomodulatory-related context, as those latter proliferations are well-known to be likely to resolve or reduce after reduction of the drugs).

**TABLE 2:** Summary of reported cases of spontaneous remission of non-Hodgkin lymphoma in the extracranial, head and neck area.

Case no	Author	Year	Sex/age	Tumour	Localization	Treatment	Other findings	Antecedent prior to remission	Time after antecedent	Follow-up
1	Burkitt et al. [29]	1966	F/4 Y	‘African lymphoma'	Maxilla	None	—	Biopsy	1 Y	2 Y
2	Grem et al. [30]	1986	F/54 Y	DLBCL	Left vallecula	None	—	Biopsy	—	4 Y
3	Poppema et al. [31]	1988	M/12 Y	Non-Burkitt's lymphoblastic lymphoma	Oropharynx	None	—	Biopsy	—	3 Y
4	Kumamoto et al. [23]	1994	F/58 Y	High grade B-cell non-Hodgkin lymphoma	Neck, axilla, inguinal	None	—	Biopsy	3 W	2 Y
5	Savarrio et al. [32]	1999	M/77 Y	CD30 ^+^ anaplastic large cell lymphoma	Soft palate	None	—	Biopsy	4 W	Relapse 12 M relapse with DLCBL, cervical region
6	Koga et al. [33]	2003	F/78 Y	DLBCL	Gingiva	None	—	Biopsy	3 W	—
7	Heibel et al. [34]	2004	–/70 Y	DLBCL	Oral mucosa	None	—	Biopsy	1 M	12 M
8	Sasaki et al. [35]	2004	F/54 Y	Primary cutaneous anaplastic large cell lymphoma	Forehead	None	—	Biopsy	1 M	—
9	Chang et al. [36]	2004	F/40 Y	DLBCL	Orbit, conjunctiva	None	—	Biopsy	5 W	4 M
10	Winhoven et al. [37]	2005	M/39 Y	CD30 ^+^ anaplastic large cell T-cell lymphoma	Upper eyelid	Mometasone furoate (0.1% cream)	—	Biopsy	2 M	2 M
11	Sakuma et al. [38]	2006	F/70 Y	MALT-lymphoma	Hard palate	None	Sjögren syndrome	Biopsy	One month after first biopsy, histological confirmation 3 months after first biopsy	38 M
12	Madan et al. [39]	2007	F/31 Y	Cutaneous peripheral T-cell lymphoma	Forehead	None	—	Biopsy	2 M	2 M
13	Ogden et al. [40]	2008	F/78 Y	B-cell lymphoma	Nose tip	Menthol 1% in aqueous cream and hydroxyzine 10 mg at night	—	Biopsy	—	—
14	Daly et al. [41]	2008	M/56 Y	T-cell lymphoma	Gingiva	None	T-cell lymphoma 14M earlier, different site	Biopsy	—	4 Y
15	Graham et al. [16]	2009	M/82 Y	Primary cutaneous B-cell lymphoma, DLBCL	Left cheek, skin	None	—	Biopsy	—	—
16	Santiago-et-Sánchez-Mateos et al. [42]	2011	M/4 Y	Primary cutaneous anaplastic large cell lymphoma	Nose tip	None	—	Biopsy	4 M	4 M
17	Tamas et al. [43]	2011	F/66 Y	DLBCL with activated B-cell like immunophenotype	Between left vallecula and tongue	None	—	Biopsy	6 M	7 Y
18	Buckner et al. [44]	2012	F/67 Y	DLBCL	Maxillary sinus	None	Pneumonia and concomitant Clostridium Difficile-infection	Concomitant infection	—	1 Y
19	Farquhar et al. [25]	2014	F/66 Y	Follicular B-cell lymphoma	Cheek	Steroids for unrelated asthma	Asthma	Steroids for unrelated asthma	2 W	—
20	Igawa et al. [45]	2015	M/80 Y	Plasmablastic lymphoma	Gingiva	None	Epstein-Barr virus	Biopsy	40 D	5 M
21	Fernandez et al. [46]	2015	M/44 Y	T-cell lymphoma	Tip of the nose	None	—	Biopsy	3 M	1 Y
22	Kaibuchi et al. [47]	2015	M/87 Y	DLCBL	Gingiva	Azithromycin (prevention of infection after biopsy)	—	Biopsy	3 W	2.5 Y
23	Miyagawa et al. [48]	2017	M/46 Y	Primary cutaneous anaplastic large cell lymphoma	Upper lip	None	—	Biopsy	—	14 M
24	Snijder et al. [49]	2019	F/88 Y	DLBCL	Cervical (level III)	None	Idiopathic pulmonary fibrosis	Biopsy	3 M	2 Y, 1 M
25	Pan et al. [50]	2019	F/55 Y	Small B cell lymphocytic cell lymphoma, IIIB	Neck, mediastinum, abdomen, inguinal	None	—	Biopsy on spleen	4.5 Y	4.5 Y
26	Flattow-Trujillo et al. [51]	2019	F/61 Y	DLBCL	Maxilla	None	Diabetes Mellitus	Biopsy	6 W	22 M
Present case	Peeters et al.	2020	M/59 Y	Follicular lymphoma	Buccal space to masticator space	None	—	Biopsy	5.5 M	6 M

F, female; M, male; DLCBL, Diffuse Large B-Cell Lymphoma; MALT, Mucosa-associated lymphoid tissue; W, week; M, month; Y, year

In follicular lymphoma, the increase of follicular helper T-cells generates a contradictory immunosuppressive tumor microenvironment, thus promoting immune escape, tumor survival and growth [14, 24]. However in this case a large number of TFH cells were associated with a spontaneous regression after 6 months. Interestingly, one may raise the question whether the particularly favorable short-term outcome in this case could be linked to the brisk clonal TFH response, albeit there is no direct proof of it.

The finding of spontaneous remission avoided unnecessary treatment, highlighting the importance of clinical alertness in follicular lymphoma. As literature lacks statistical data regarding relapse after spontaneous remission of lymphoma and shows cases of relapse after spontaneous remission, close follow-up is strongly recommended. Moreover, the rare nature of the regression commands caution in interpreting this case report; postponing treatment or deviating from general treatment principles should be avoided.

## Conclusion

It is important for the clinician, pathologist, radiologist and hematologist to be aware of the non-specific presentation of lymphoma. Mimicking other malign and benign lesions, lymphoma should be considered in the differential diagnosis when a tumor mass presents in the head and neck region. Moreover, the importance of clinical alertness is highlighted in this case, as a spontaneous remission was found unexpectedly, avoiding unnecessary treatment and implying close follow-up.

## Data Availability

The original contributions presented in the study are included in the article/Supplementary Material, further inquiries can be directed to the corresponding author.

## Appendix 1: PDF VECTRA

3D-view of the patient at the moment of first presentation, using VECTRA^®^ H1 (Canfield Scientific Europe, Utrecht, Netherlands) (Supplementary Data Sheet 1). The swelling presented at the infraorbital region (it is observed best in cranial or caudal view). The zone of hypo-aesthesia is demarcated.

## Appendix 2: Patient Timeline

MRI, Magnetic Resonance Imaging; OMFS-Department, Oral and Maxillofacial Surgery Department; 18F-FDG PET/CT, 18F-fluorodeoxyglucose Positron Emission Tomography/Computed Tomography.
