# Supplementary figures and images for: Case Report: Spontaneous Remission of an Infraorbital Follicular B-Cell Lymphoma: Case Report and Review of the Literature
Source: Pathol Oncol Res. 2021 Apr 8;27:642433. doi: 10.3389/pore.2021.642433 (PMC8262163; doi:10.3389/pore.2021.642433)

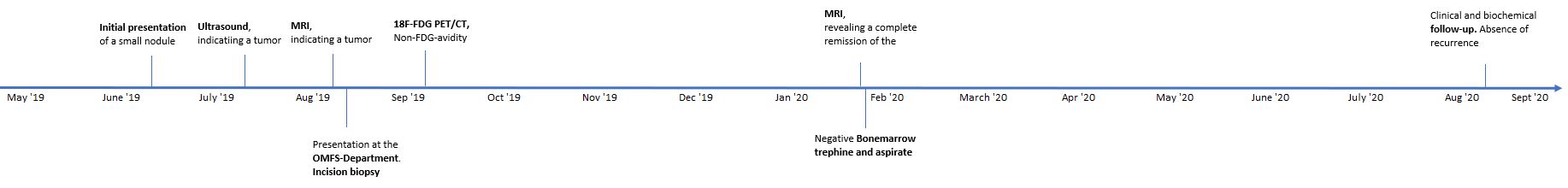

Supplement: Supplementary file 2 [file Image1.JPEG]
